# Anti-leukaemia activity as a bystander effect of graft-versus-host reactions.

**DOI:** 10.1038/bjc.1976.61

**Published:** 1976-04

**Authors:** P. R. Johnson, P. Hersey

## Abstract

The production of graft-versus-host (GVH) reactions in (PVGc X Wistar) F1 hybrids by the transfer of PVGc spleen cells resulted in significant resistance of these recipients to a subsequent challenge with the PVGc leukaemia. Protection was markedly dependent on dose and timing of allogeneic cell transfer and was abrogated by irradiation of the cells prior to transfer. GVH activity was shown to be a prerequisite for induction of the protective effect but was equally effective when produced by the transfer of Wistar spleen cells in place of PVGc cells. These points, plus the fact that invitro investigations of possible immune mechanisms failed to demonstrate cytotoxic immunity in treated rats, suggested a nonspecific "bystander" effect as the mechanism of protection. The implications of such a mechanism are discussed.


*Br. J. Cancer* (1976) **33**, 370

### ANTI-LEUKAEMIA ACTIVITY AS A BYSTANDER EFFECT OF GRAFT-VERSUS-HOST REACTIONS

P. R. JOHNSON AND P. HERSEY

*From the Immunology Unit, Department of Bacteriology, University of Sydney, N.S.W., Australia*

Received 31 October 1975 Accepted 12 December 1975

**Summary.—The production of graft-versus-host (GVH) reactions in ($PVG_C$ × Wistar) $F_1$ hybrids by the transfer of $PVG_C$ spleen cells resulted in significant resistance of these recipients to a subsequent challenge with the $PVG_C$ leukaemia. Protection was markedly dependent on dose and timing of allogeneic cell transfer and was abrogated by irradiation of the cells prior to transfer. GVH activity was shown to be a prerequisite for induction of the protective effect but was equally effective when produced by the transfer of Wistar spleen cells in place of $PVG_C$ cells.**

**These points, plus the fact that *in vitro* investigations of possible immune mechanisms failed to demonstrate cytotoxic immunity in treated rats, suggested a non-specific " bystander " effect as the mechanism of protection. The implications of such a mechanism are discussed.**

THE DEMONSTRATIONS of increased immune responsiveness following allogeneic cell interactions (Hamilton, 1973; Katz, 1972) raised the question whether GVH reactions could have a similar effect on immune responses against tumours. Some evidence for this comes from several reports. Medzihradsky (1966) showed that the induction of a GVH reaction in (Lewis × AVN) $F_1$ rats given the Walker tumour resulted in prolonged survival times and a decrease in the number of lethal takes of the tumour. Similar results were obtained with a methylcholanthrene-induced tumour when a GVH reaction was induced simultaneously with tumour inoculation (Medzihradsky, Konikova and Novotna, 1973).

In guinea-pigs the transfer of allogeneic lymphoid cells has been shown to produce resistance to a subsequent inoculum of leukaemia cells. This resistance was manifested by striking prolongation of survival times and in some animals which survived indefinitely, resistance to a second chellenge with leukaemia was demonstrable. The effect was apparently dependent on a GVH reaction but attempts to determine the mechanisms involved were unsuccessful (Ellman *et al.*, 1972; Katz *et al.*, 1972).

In many of the studies involving anti-tumour effects produced by the transfer of allogeneic cells, it has been difficult to divorce any direct cytotoxic effects the allogeneic cells may have on the tumour from the effects of interaction of allogeneic cells with those of the host. We have therefore chosen a GVH model in which the donor and recipient are histocompatible with the tumour and where the effects observed can presumably be attributed solely to the effects of the allogeneic interaction of lymphoid cells. We report here our results showing that interaction of lymphoid cells in a GVH reaction in these circumstances can result in marked inhibition of growth of a highly malignant leukaemia in rats. We have further characterized the nature of this anti-tumour activity and present the results of initial studies designed to reveal the mechanism of this effect.

Correspondence to Dr P. Hersey, Kanematsu Institute, Sydney Hospital, Sydney, N.S.W., Australia.

### MATERIALS AND METHODS

*Animals.*—8–10 week old rats of the inbred $PVG_c$ strain were from the MRC laboratories, Carshalton, Surrey, U.K. Wistar strains and the $F_1$ hybrid of this combination with the $PVG_c$ were from the University of Sydney animal station.

*Tumour.*—The leukaemia used in these studies was the $PVG_c$ lymphoma kindly supplied by Dr Bruce Roser of the Department of Pathology, University of Sydney. The tumour has a marked leukaemic phase concurrent with lymph node involvement. A highly virulent form of this tumour, obtained by repeated passage in syngeneic animals, was used in these studies. In this form only minimal involvement of lymph nodes is seen and the disease is predominantly that of a rapidly fatal leukaemia. An i.p. dose of $10^4$ leukaemic cells was used in all experiments and produced leukaemia within about 14 days, and death no longer than 10 days after this. No evidence for resistance on the part of susceptible rats ($PVG_c$ and $PVG_c$ hybrids) has yet been demonstrated (Hersey, 1973*a*; 1973*b*).

Tumour cell suspensions were obtained from the peritoneal cavity of previously i.p. infected animals by lavage through a wide-bore perforated needle, with Eagle's minimal essential medium (MEM) (Microbiological Assoc., Inc., U.S.A., Cat. No. 12–92) containing Penicillin (100 units/ml) and Streptomycin (100 $\mu$g/ml).

To determine the day of onset of leukaemia, sequential blood counts of inoculated animals were made on a model D Coulter Counter (Coulter Electronics Ltd., Dunstable, Beds., England). Rats reaching a count higher than $30 \times 10^6$/ml of whole blood on two successive days were classed as leukaemic, since all such rats showed progressive increases in blood count from this point on.

*Neonatal induction of tolerance.*—$PVG_c$ rats less than 24 h old were rendered neonatally tolerant of ($PVG_c \times$ Wistar) $F_1$ by the injection of $8 \times 10^7$ hybrid bone marrow cells via the intracardiac route, as described by Grazer (1958).

Tolerance was assessed by a popliteal lymph node weight assay, described by Ford, Burr & Simonsen (1970) for the *in vivo* detection of GVH reactivity. In this assay $5 \times 10^6$ lymphoid cells were injected into the footpads of $F_1$ hybrid rats and 7 days after this, the popliteal lymph nodes were excised and weighed. Lymphoid cells from successfully tolerized $PVG_c$ rats produced no increase in node weight while normal $PVG_c$ cells produced a 4- to 5-fold increase in weight.

### In vitro $^{51}$*Cr release assays*

$^{51}$*Cr labelling of target cells.*—Approximately $3 \times 10^6$ tumour cells in 1 ml of MEM supplemented with 10% Foetal Bovine Serum (FBS) (Australian Laboratory Services, Batch 28) were incubated with 100 $\mu$Ci of $Na_2\,^{51}CrO_4$ (Radiochemical Centre, Amersham, Bucks., U.K.) for 2 h at 37°C. The cells were washed twice in 25 ml of MEM and resuspended in MEM + 10% FBS buffered with 20 mM of Hepes.

*Antibody-dependent (AD) cell-mediated cytotoxicity.*—Assay of rat sera for this activity was carried out as previously described (Hersey, 1973*b*) using human effector cells obtained by Hypaque/Ficoll separation of peripheral blood. $6 \times 10^5$ effector cells in 0·2 ml were used in each assay and $2 \times 10^4$ target cells were added in 0·2 ml. Test sera from rats were added in serial concentrations in duplicate in a volume of 0·05 ml. The total volume of 0·45 ml was incubated in capped round-bottomed tubes for 6 h at 37°C. After this the cells were sedimented by centrifugation and 0·2 ml of each supernatant was transferred to adjacent tubes for counting.

*Direct cell-mediated cytotoxicity.*—Spleen cells were assayed for direct cytotoxicity activity after depletion of macrophages by glass absorption (Hersey, 1973*a*). Absorbed spleen cells at concentrations of $3 \times 10^6$, $1 \times 10^6$ and $3 \times 10^5$ in 0·5 ml were added to target cells ($2 \times 10^4$ in 0·5 ml). Incubation was for 6 h at 37°C in capped flat-bottomed tubes. After incubation the cells were centrifuged and 0·5 ml of supernatant transferred to adjacent tubes for counting. All counting was done on a Wallac Gamma Sample Counter.

Percent $^{51}$Cr release was calculated as follows:

$$\%\ ^{51}\text{Cr release} = \frac{a \times 2{\cdot}25}{a + b} \times 100$$

where $a$ = activity in supernatant tube and $b$ = activity in tube with cell pellet and remaining supernatant. For the direct cytotoxic assay, the factor was 2 in place of 2·25.

In vitro *assay for cytostatic serum factors.*—Spontaneous $^{3}H$-thymidine uptake by leukaemic cells was assessed by short-term culture of $3 \times 10^{6}$ cells in a volume of 1 ml of Dulbecco's modified Eagle's medium (DEM), supplemented with 10% FBS, penicillin and streptomycin. Cultures were pulsed for 4 h with 2 $\mu$Ci of isotope at specific activity of 5 Ci/mM (Radiochemical Centre, Amersham) 2 h after the cultures were set up. Test and control serum was added in varying dilutions to these tubes at the time of dispensing to determine whether such sera could produce a decrease in isotope uptake. Harvesting and counting were carried out by standard trichloroacetic acid extraction and $\beta$ scintillation counting procedures.

*Experimental protocol.*—Spleen cells were taken from parental $PVG_c$ rats (except where otherwise indicated) approximately 2 months old, and injected slowly i.v. into $F_1$ rats 6–8 weeks of age at the times indicated in the text, in relation to the tumour inoculum of $10^{4}$ cells given i.p. on Day 0. Controls in each experiment were rats given tumour only or in some experiments rats given tumour and $F_1$ spleen cells at the same doses as the $PVG_c$ spleen cells. Leukaemia onset was determined by sequential count of blood from the tail veins.

*Statistical analysis.*—Wilcoxon's two sample rank test was employed to assess the significance of variations in the day of leukaemia onset between groups of 5–6 rats. Probability levels of 5% were chosen as significant.

*Irradiation.*—Spleen cells were irradiated by a $^{60}Co$ source at a distance of 12 inches in a plane vertical to the beam axis on a rotating platform. Dose rate was 50 rad/min.

### RESULTS

*The protective effect of $PVG_c$ spleen cells.*—(Figure 1.) To determine whether $PVG_c$ spleen cells could produce protection from leukaemia in ($PVG_c \times$ Wistar) $F_1$ rats, spleen cells in varying numbers were transferred i.v. to $F_1$ rats 6 days before these rats received an i.p. inoculation of $10^{4}$ tumour cells. The time of onset of leukaemia in these rats was determined and compared with that of a group which had received $10^{8}$ syngeneic ($F_1$) cells on Day $-6$.

Rats pretreated with $3 \times 10^{7}$ or $10^{8}$ $PVG_c$ spleen cells showed significant delays in leukaemia onset when compared with the control group ($P < 0{\cdot}01$). Recipients of $3 \times 10^{8}$ $PVG_c$ spleen cells showed no delay in onset.

FIG. 1.—Results show % of $F_1$ rats in each group with leukaemia on sequential days after leukaemia inoculation. Group 1 rats received $10^{8}$ $F_1$ spleen cells on Day $-6$; groups 2, 3 and 4 received $3 \times 10^{8}$, $3 \times 10^{7}$ and $10^{8}$ $PVG_c$ spleen cells respectively, on Day $-6$. All rats were inoculated with $10^{4}$ leukaemic cells on Day 0.

FIG. 2.—$F_1$ rats received $10^8$ $PVG_c$ spleen cells at varying intervals with respect to tumour inoculation on Day 0. Group 1, Day 0; 2, Day −21; 3, Day +6; 5, Day −14; 6, Day −6. Group 4 received no spleen cells.

*Time dependence of the effect.*—(Fig. 2.) The effect of variation in the time of cell transfer was assessed by transfer of a uniform dose of $10^8$ $PVG_c$ spleen cells into $F_1$ hybrids on Days −21, −14, −6, 0 or +6, and $10^4$ tumour cells on Day 0. It was found that cells transferred on Day −6 provided significant protection ($P < 0{\cdot}01$). All other times of cell transfer were ineffective.

Despite the demonstration that cell transfers on Days 0 and +6 were ineffective, when $10^8$ $PVG^c$ spleen cells were given to a group of animals on Days −6, 0 and +6 an additive protective effect was noted compared to that of a group receiving spleen cells on Day −6 only (Groups 2 and 3 in Table I, $P < 0{\cdot}05$).

*The effect of irradiation* (1000 *r*) *of $PVG_c$ spleen cells before transfer.*—(Table I.) Using the optimal conditions for protection, a transfer of $10^8$ $PVG_c$ spleen into $F_1$ hybrids produced a significant delay in leukaemia onset. When, however, such cells were pretreated with 1000 r, no protective effect was observed. Since it was felt that irradiated cells might be more rapidly cleared from the circulation and hence be prejudiced in their protective potential, a group of rats received 3 doses of $10^8$ irradiated cells on Days −6, 0 and +6 (with leukaemia challenge on Day 0). This produced a significant delay in onset compared with recipients of leukaemia cells only ($P < 0{\cdot}05$). However, the degree of protection achieved with 3 doses of irradiated cells was significantly less than that produced by either 1 or 3 doses of unirradiated cells ($P < 0{\cdot}01$).

*The effect of Wistar spleen cells on leukaemia onset.*—(Table II.) To test whether protection was due to immunization of $F_1$ animals against antigens cross-reacting between $PVG_c$ spleen cells and leukaemia cells (*i.e.* strain specific), Wistar spleen cells were used instead of $PVG_c$ spleen cells. As shown in Table II, when $10^8$ Wistar spleen cells were given on Day −6 in relation to the tumour inoculum, a significant delay in onset of leukaemia was observed ($P < 0{\cdot}01$) suggesting that allogeneic in-

TABLE I.—*The Effect of Irradiation of Transfused Syngeneic Donor Cells on their Anti-leukaemic Effect*

| Group | Day of leukaemia onset | $P$ value vs. Gp. 1 |
|---|---|---|
| 1. Tumour only | 13, 13, 14, 14, 14, 14 | — |
| 2. $10^8$ cells | 16, 16, 16, 17, 17, 18 | $<0{\cdot}01$ |
| 3. $10^8$ cells × 3* | 18, 19, 19, 20, 20, 20† | $<0{\cdot}01$ |
| 4. $10^8$ irrad. cells | 14, 14, 15, 15, 15, 15 | NS |
| 5. $10^8$ irrad. cells × 3* | 14, 14, 15, 15, 15, 16 | $<0{\cdot}05$ |

* On Days −6, 0, and +6 in relation to inoculum of $10^4$ tumour cells on Day 0.

† Greater than Group 5 ($P < 0{\cdot}02$).

TABLE II.—*Effect of Transfer of Allogeneic Donor Cells on Leukaemia Onset*

| Group | Day of leukaemia onset | $P$ value 1 vs. 2 |
|---|---|---|
| 1. Tumour only | 15, 15, 15, 16, 16 | — |
| 2. $10^8$ Wistar spleen cells Day −6 | 17, 18, 18, 19, 24, 25 | <0·01 |

teraction of the cells was responsible for the effect.

*The effect of $F_1$ spleen cells on leukaemia onset in $PVG_c$ rats.*—Since protection was apparently dependent on an allogeneic cell interaction it was necessary to assess whether protection could be provided by the interaction of Parent and $F_1$ cells in a HVG situation. When $10^8$ $F_1$ hybrid spleen cells were transferred to $PVG_c$ rats 6 days before leukaemic inoculation there was no significant difference between the time of leukaemia onset in these rats and in rats which received tumour cells only.

*The effect of tolerant $PVG_c$ spleen cells.*—(Figure 3.) The dependence of protection upon a GVH reaction was further investigated by assessing the effect of spleen cells from $PVG_c$ rats which had been rendered neonatally tolerant of ($PVG_c \times$ Wistar) $F_1$ and shown to be incapable of inducing a GVH response *in vivo.*

Rats given $10^8$ tolerant spleen cells on Day −6 and $10^4$ leukaemia cells on Day 0, failed to demonstrate a delay in onset of leukaemia, although $10^8$ normal cells produced significant delay ($P < 0{\cdot}01$). As a further control in this experiment a group of rats received $10^8$ " non-tolerant " cells from $PVG_c$ rats in which tolerance induction had failed and which were therefore able to produce GVH reactions in the *in vivo* GVH assay. As shown in Fig. 3 recipients of such cells showed a significant degree of delay, similar to that produced by normal $PVG_c$ cells.

*AD cell-mediated cytotoxicity.*—(Figure 4.) Groups of $F_1$ rats were given $10^8$ $PVG_c$ spleen cells on Day −6 and serum samples taken on Days 0, +6 and +12 in relation to the tumour inoculum. Samples from 3 individual rats were assayed at these time periods for evidence of AD cell-mediated cytotoxic activity and as shown in Fig. 4 no evidence of this

FIG. 3.—All groups of $F_1$ rats received $10^4$ $PVG_c$ leukaemia cells on Day 0. In addition, on Day −6, group 1 rats received $10^8$ "tolerant" $PVG_c$ spleen cells; group 3, $10^8$ "unsuccessfully tolerized" $PVG_c$ spleen cells (see text); group 4, $10^8$ normal $PVG_c$ spleen cells. Group 2 received no spleen cells.

FIG. 4.—AD cell-mediated cytotoxic activity of sera from $F_1$ rats receiving $10^8$ $PVG_C$ spleen cells on Day −6 and $10^4$ $PVG_C$ leukaemia cells on Day 0. Target cells: $^{51}Cr$-labelled $PVG_C$ leukaemia cells. Sera at (a) Day 0; (b) Day +6; (c) Day +12; (d) from cytotoxic control (see text). Each point represents the mean results of 3 different sera assayed in duplicate.

activity was found. Sera from Wistar rats immunized with $10^8$ $PVG_C$ lymphoma cells were included in the assay as a positive control and indicate the sensitivity of the assay.

*Direct cell-mediated cytotoxicity.*—(Fig. 5.) Direct cell killing was assessed in macrophage-depleted spleen populations from $F_1$ rats treated with $10^8$ $PVG_C$ spleen cells on Day −6. Rats were sacrificed at Days 0, +6 and +14 and their spleens assayed for the presence of cytotoxic activity against the leukaemia cells. No significant levels of cytotoxicity were demonstrated at these times. Graph (d) represents the level of cytotoxicity developed in Wistar rats inoculated with $PVG_C$ lymphoma 5 days before assay.

*Cytostatic serum factors.*—Leukaemic cells were found to incorporate spontaneously a reproducible level of $^3H$-thymidine under short-term culture conditions. Sera from experimental rats were added in varying dilutions to see whether they could exert an inhibitory effect on isotope uptake. When the effect of such sera was compared with the effect of sera from normal rats, no significant depression of isotope uptake could be demonstrated at any time during the GVH protocol. It was, however, found that all sera from normal or test rats had a markedly inhibitory effect on isotope uptake by the leukaemic cells.

### DISCUSSION

The use of allogeneic lymphoid cells in immunotherapy of leukaemia has been reported by several workers (Woodruff and Nolan, 1963; Mathé *et al.*, 1967; Boranić, 1968; Britton, 1972; Bortin, 1974). The anti-leukaemia effect in these studies has generally been regarded as an example of adoptive immunotherapy, resulting from histocompatibility differ-

FIG. 5.—Lymphocyte cytotoxicity assay of splenic cells from $F_1$ rats undergoing GVH protocol and tumour inoculation. Spleens taken at (a) Day 0; (b) Day +6; (c) Day +14; (d) cyctotoxic control (see text).

ences between donor lymphoid cells and the tumour of the recipient. In contradistinction, the data presented here demonstrate an anti-leukaemic effect in a GVH reaction in which both donor and recipient are syngeneic with the leukaemia. The anti-leukaemic effect, in terms of delay in the time of leukaemia onset, was equivalent to a 3–4 log reduction in the size of the initial $10^4$ leukaemia cell inoculum even though both $PVG_c$ and the $F_1$ hybrid are incapable of rejecting inoculations of very small numbers of these tumour cells (Hersey, 1973c).

Over the past few years several immunological effects of allogeneic cell interactions have been described, other than the anti-tumour effects described here. Increased antibody production after primary and secondary antigenic challenge has been described and referred to as the " allogeneic effect " (Katz, 1972; Hamilton, 1973). Abrogation of tolerance has also been described under similar conditions by McCullagh (1970). Similarities of the anti-tumour effect to these later phenomena are apparent. The timing of the GVH reaction in relation to the challenge with tumour cells or antigen has been found critical in all studies and coincides with the time of peak GVH activity. Similarly the dosage of cells given to the recipients has also been shown to be important so that, in our own studies, if the parental cell inoculum was either too large or too small the effect was abrogated. Also, as in our own studies, irradiation of the donor cells rendered them much less effective in producing the described effect suggesting that viable cells capable of multiplication were needed.

In view of the similarities noted with these other described effects of allogeneic cell interactions, the question arises to what extent they can be implicated in the anti-tumour effect described in this model. To answer this question we have carried out *in vitro* studies to detect either direct cell-mediated or AD cell-mediated immunity. Our failure to find any evidence of cytotoxic immunity by the assays argues against specific immune mechanisms being involved but cannot be regarded as definitely excluding them.

The assays used have been shown by previous studies (Hersey, 1973*a*, *b*), and by the controls used in the present study to be sensitive in detection of these mechanisms after allogeneic immunizations but could, nevertheless be ineffective in the semi-syngeneic model used here.

Another possible mechanism of the anti-tumour effect considered in this study was that the $F_1$ recipients recognized antigens on the $PVG_c$ spleen cells and were in effect being immunized against similar antigens on the $PVG_c$ leukaemia. Although this contradicts classical transplantation dogma, the concept of anti-parental responses receives some support from the literature (Cudcowicz and Stimpfling, 1964; Field, Cauchi and Gibbs, 1967; Ramseier and Lindenmann, 1969).

Several of the experiments described in this study argue strongly against this possibility. Firstly it was shown that $PVG_c$ cells tolerant to the $F_1$ recipient did not give rise to any anti-tumour effects. As it seems likely that the tolerant cells have the same antigens as non-tolerant cells this argues against the effect being a result of recognition of parental antigens. Similarly the experiments using irradiated spleen cells can be interpreted in the same way. Even when 3 times the dose of irradiated cells was given the anti-leukaemic effect was still much less than that resulting from a single dose of unirradiated spleen cells. Secondly the kinetics of the protection argues against an immunization effect, in that cells given 14 or 21 days before could be expected to provide a similar immunization to that at 6 days. For further evidence against this mechanism the experiment using Wistar spleen cells can be cited. If immunization against $PVG_c$ spleen cells was involved, then Wistar spleen cells could be expected to be less effective than $PVG_c$ spleen cells. This was found not to be the case and equal protection was afforded by Wistar spleen cells.

We therefore consider the most likely explanation for the observed anti-tumour effect is that it results from non-specific cytotoxic activity generated during the course of the GVH reaction. Non-specific cytotoxic effects of GVH activity have been suggested by the *in vivo* studies of Elkins and Guttman (1968) and Streilein and Billingham (1970). *In vitro* analyses of such effects by Singh, Sabbadini and Sehan (1971, 1972) showed that non-specific cytotoxic effects were generated 4–8 days after parental cell transfer and were mediated by host cells. More recent demonstrations of similar effects were made by Britton (1974) who found evidence of *in vitro* cytotoxicity against syngeneic lymphoma cells in mice at the time of peak GVH activity.

If our interpretation is correct it is clear that " non-specific " cytotoxic mechanisms may play an effective role against tumour cells *in vivo* and may underlie the protective effect seen in several immunotherapy procedures involving transfer of allogeneic cells. If so, the measurement of non-specific cytotoxicity against tumour cells may be of equal importance to that of specific immune mechanisms in the evaluation of host resistance to tumours and in the development of immunotherapy procedures.

This work was supported by the University of Sydney Cancer Fund. We wish to thank Evelyn Adams for her able technical assistance.

### REFERENCES

BORANIĆ, M. (1968) Transient Graft-versus-host Reaction in the Treatment of Leukemia in Mice. *J. natn. Cancer Inst.*, **41**, 421.

BORTIN, M. M. (1974) Graft Versus Leukaemia. *Clin. Immunobiol.*, **2**, 287.

BRITTON, S. (1972) When Allogeneic Mouse Spleen Cells are Mixed *in vitro* the T-cells Secrete a Product which Guides the Maturation of B-cells. *Scand. J. Immunol.*, **1**, 89.

BRITTON, S. (1974) Graft-versus-host Reactions, Tumour Specific Immunity and Self Tolerance. In: *Immunological Tolerance—Mechanisms and Potential Therapeutic Applications.* Eds Katz, D. H. & Benacerraf, B. New York: Adademic Press. p. 319.

CUDCOWICZ, G. & STIMPFLING, J. H. (1964) Hybrid Resistance to Parental Marrow Grafts: Association with the K Region of H-2. *Science, N.Y.*, **144**, 1339.

ELKINS, W. L. & GUTTMAN, R. D. (1968) Pathogenesis of a Local Graft-versus-host Reaction: Immunogenicity of Circulating Host Leukocytes. *Science, N.Y.*, **159**, 1250.

ELLMAN, L., KATZ, D. H., GREEN, I., PAUL, W. E. & BENACERRAF, B. (1972) Mechanisms Involved in the Anti-leukaemic Effect of Immunocompetent Allogeneic Lymphoid Cell Transfer. *Cancer Res.*, **32**, 141.

FIELD, E. O., CAUCHI, M. N. & GIBBS, J. E. (1967) The Transfer of Refractoriness to G-V-H Disease in $F_1$ Hybrid Rats. *Transplantation*, **5**, 241.

FORD, W. L., BURR, W. & SIMONSEN, M. (1970) A Lymph Node Weight Assay for the GVH Activity of Rat Lymphoid Cells. *Transplantation*, **10**, 258.

GRAZER, F. M. (1958) Technique for Intravascular Injection and Bleeding of Newborn Rats and Mice. *Proc. Soc. exp. Biol. Med.*, **99**, 407.

HAMILTON, J. A. (1973) Allogeneic Reactions and Antibody Production. *Curr. Titles Immun. Transplant. Allergy*, **1**, 362.

HERSEY, P. (1973*a*) Thymus-dependent Cytotoxic Lymphocytes in the Rat. *Eur. J. Immun.*, **3**, 748.

HERSEY, P. (1973*b*) New Look at Antiserum Therapy of Leukaemia. *Nature, New Biol.*, **244**, 22.

HERSEY, P. (1973*c*) The Protective Effect of Antisera Against Leukaemia *in vivo*.—A Reappraisal. *Br. J. Cancer*, **28** (Suppl. 1), 16.

KATZ, D. H. (1972) The Allogeneic Effect on Immune Responses. Model for Regulatory Influences of T Lymphocytes on the Immune System. *Transpl. Rev.*, **12**, 141.

KATZ, D. H., ELLMAN, L., PAUL, W. E., GREEN, I. & BENACERRAF, B. (1972) Resistance of Guinea Pigs to Leukemia Following Transfer of Immunocompetent Allogeneic Lymphoid Cells. *Cancer Res.*, **32**, 133.

MCCULLAGH, P. (1970) The Abrogation of Sheep Erythrocyte Tolerance in Rats by Means of the Transfer of Allogeneic Lymphocytes. *J. exp. Med.*, **132**, 916.

MATHÉ, G., SCHWARZENBERG, L., AMIEL, J. L., SCHNEIDER, M., CATTAN, A. & SCHLUMBERGER, J. R. (1967) The Role of Immunology in the Treatment of Leukaemias and Hematosarcomas. *Cancer Res.*, **27**, 2542.

MEDZIHRADSKY, J. (1966) Modification of Tumour Homograft Immunity During the Graft-versus-host Reaction. *Neoplasma*, **13**, 223.

MEDZIHRADSKY, J., KONIKOVA, E. & NOVOTNA, E. (1973) On the Graft-versus-host Nature of the Allogeneic Effect in a Tumor Isograft System. *Neoplasma*, **20**, 607.

RAMSEIER, H. & LINDENMANN, J. (1969) $F_1$ Hybrid Animals: Reactivity Against Recognition Structures of Parental Strain Lymphoid Cells. *Path. Microbiol.*, **34**, 379.

SINGH, J. N., SABBADINI, E. & SEHAN, A. H. (1971) Non Specific Cytotoxicity in Graft-versus-host Reactions. *Transplantation*, **11**, 475.

SINGH, J. N., SABBADINI, E. & SEHAN, A. H. (1972) Cytoxicity in Graft-versus-host Reactions. I. Role of Donor and Host Spleen Cells. *J. exp. Med.*, **136**, 39.

STREILEIN, J. W. & BILLINGHAM, R. E. (1970) An Analysis of Graft-versus-host Disease in Syrian Hamsters. II. The Epidermolytic Syndrome: Studies on its pathogenesis. *J. exp. Med.*, **132**, 181.

WOODRUFF, M. F. A. & NOLAN, B. (1963) Preliminary Observations on Treatment of Advanced Cancer by Injection of Allogeneic Spleen Cells. *Lancet*, ii, 426.


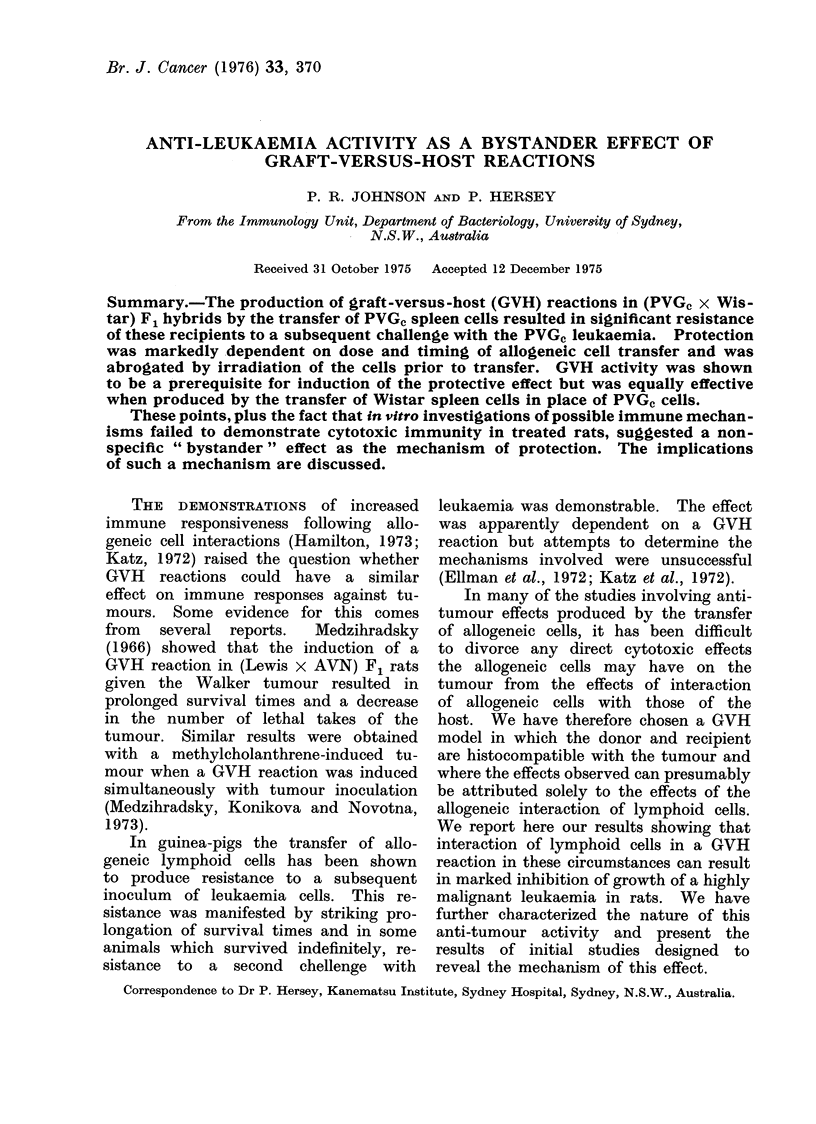

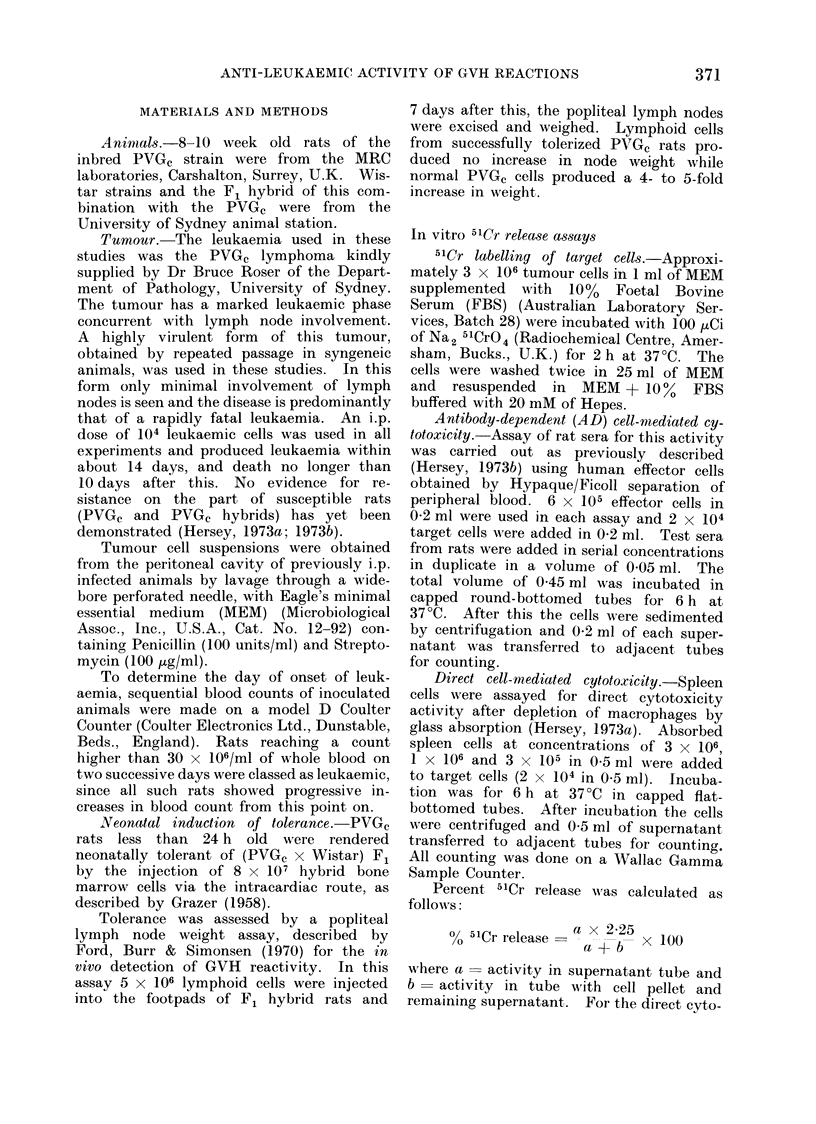

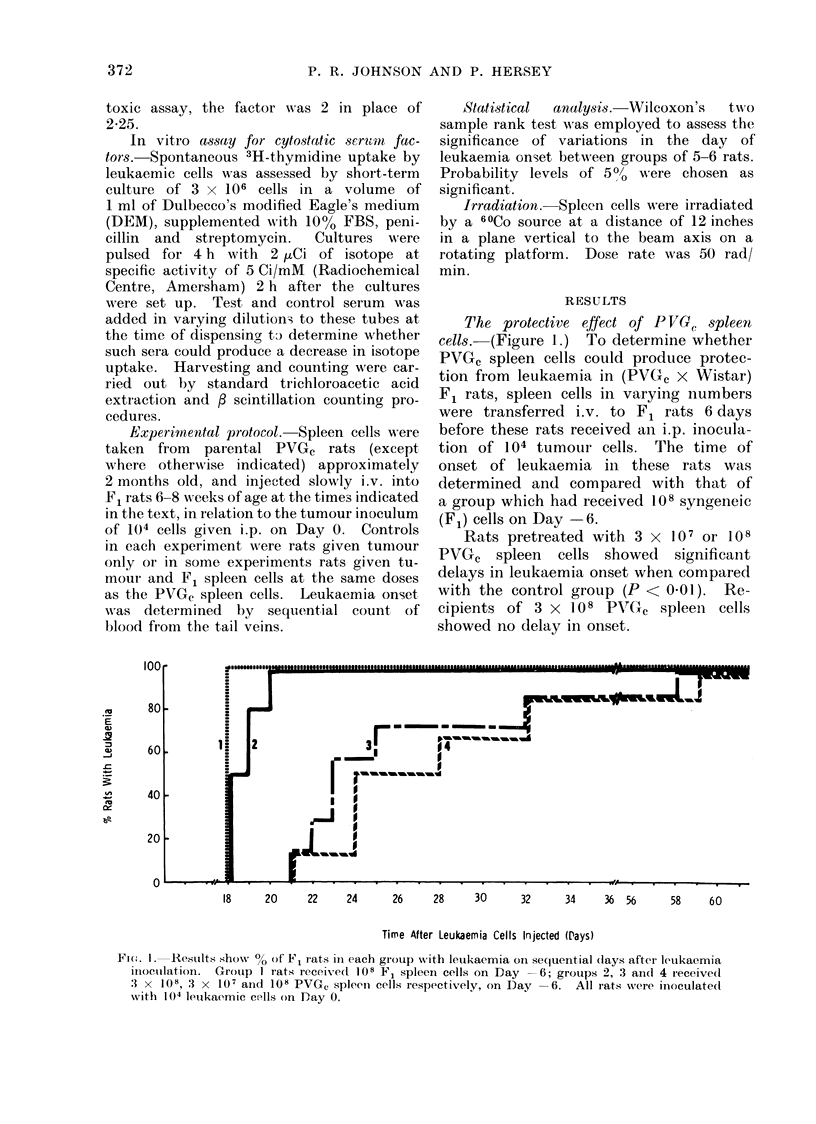

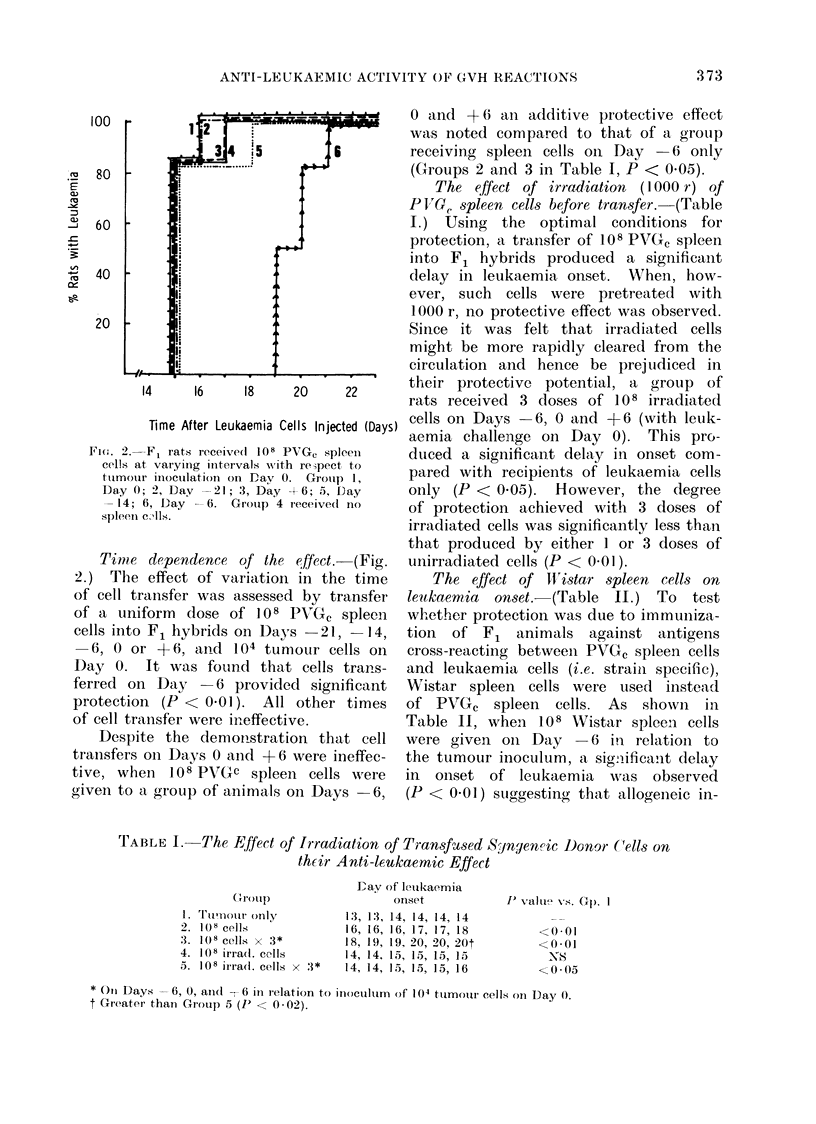

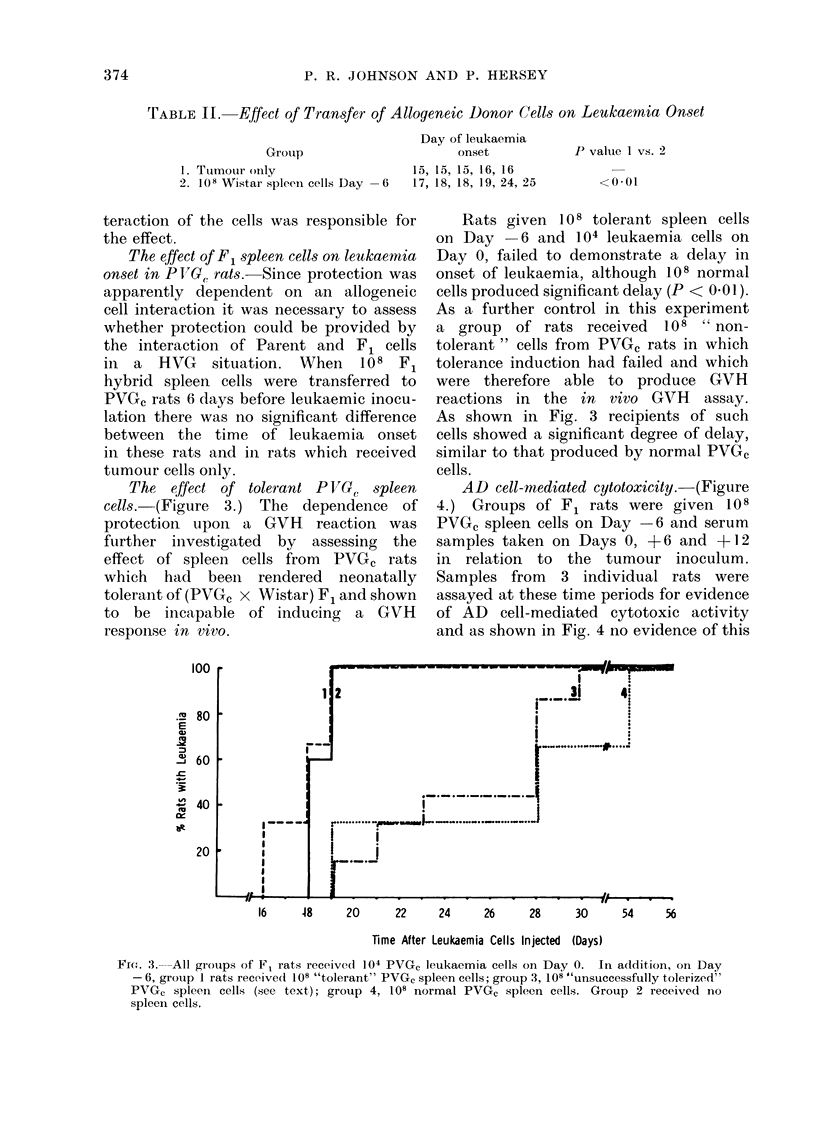

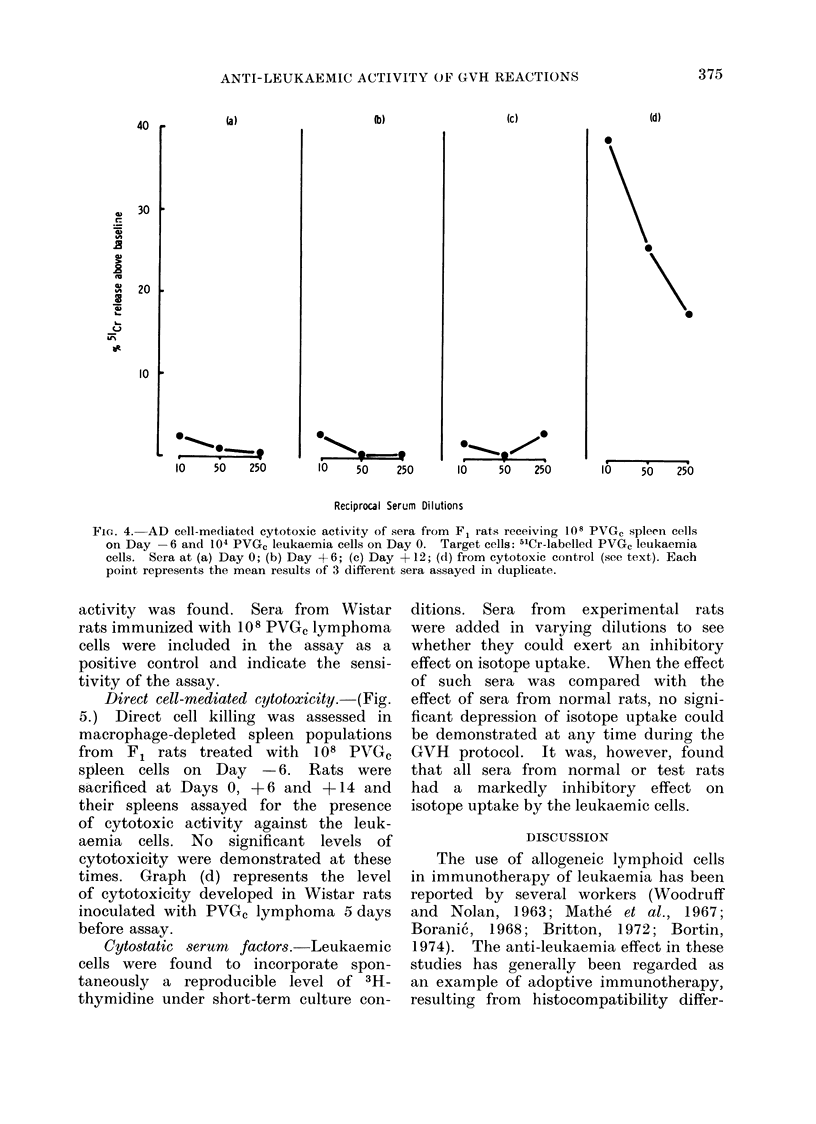

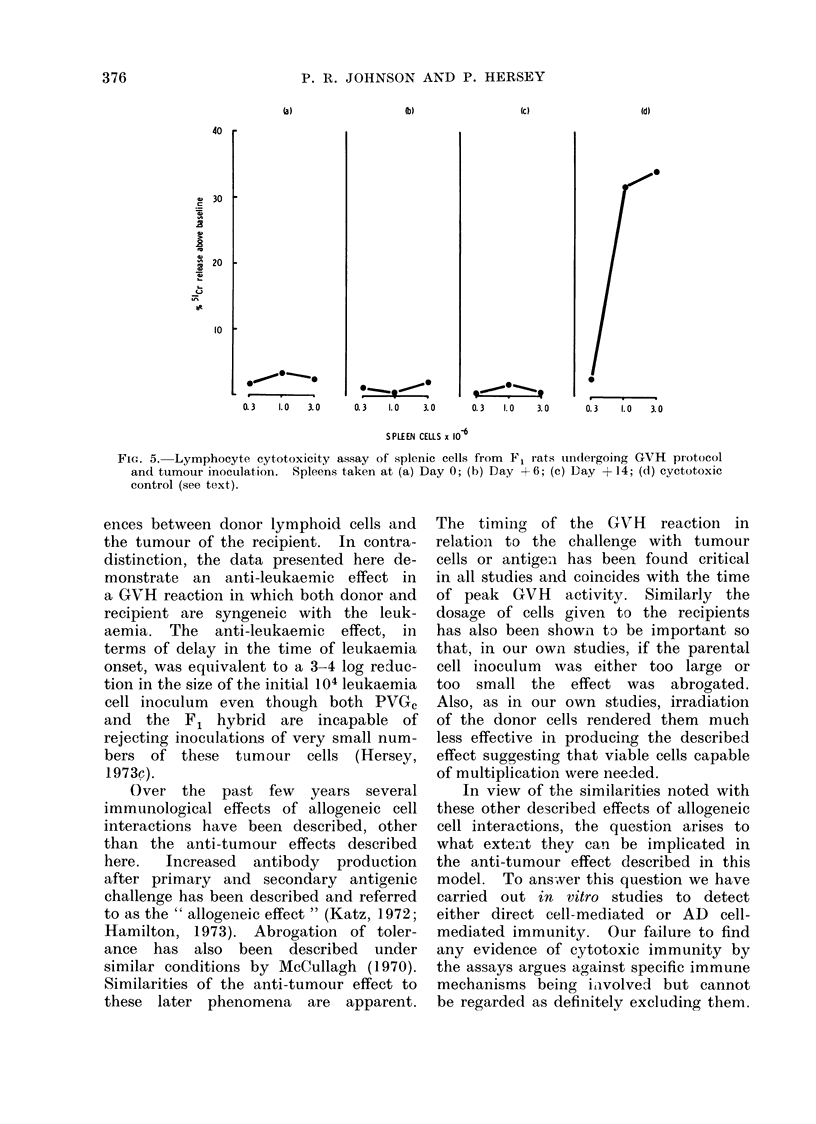

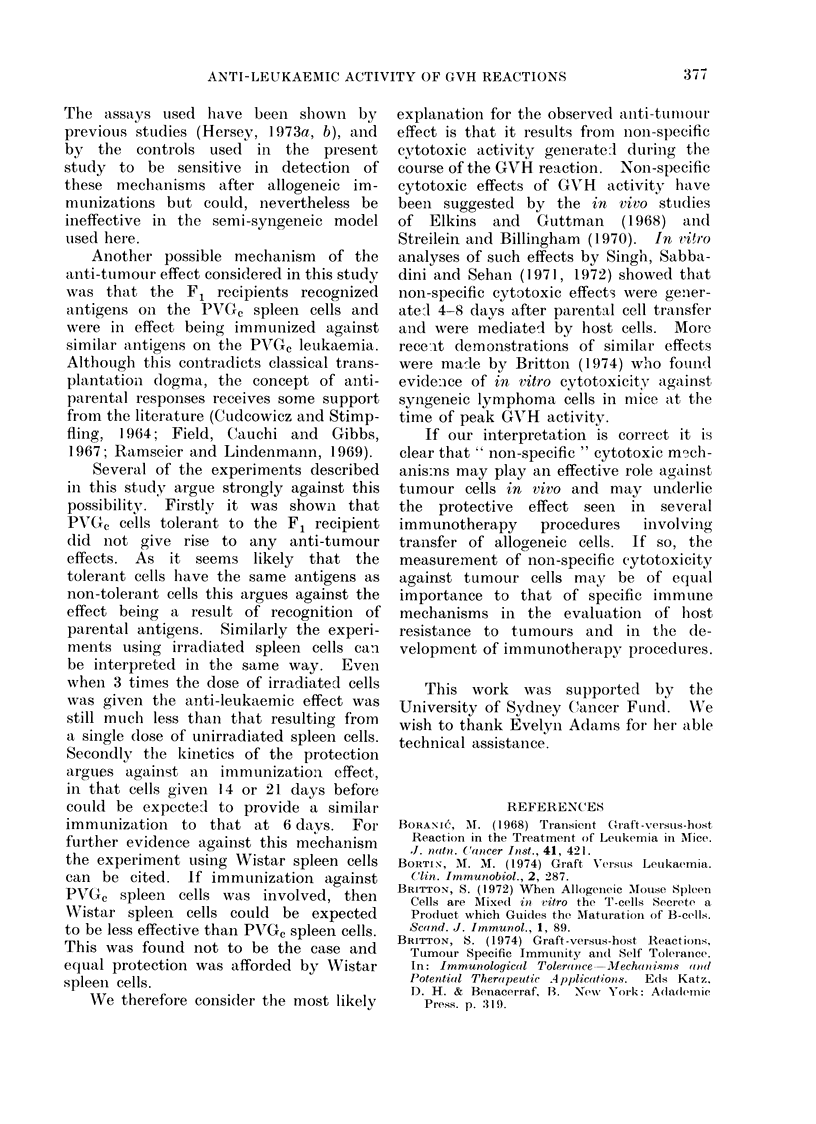

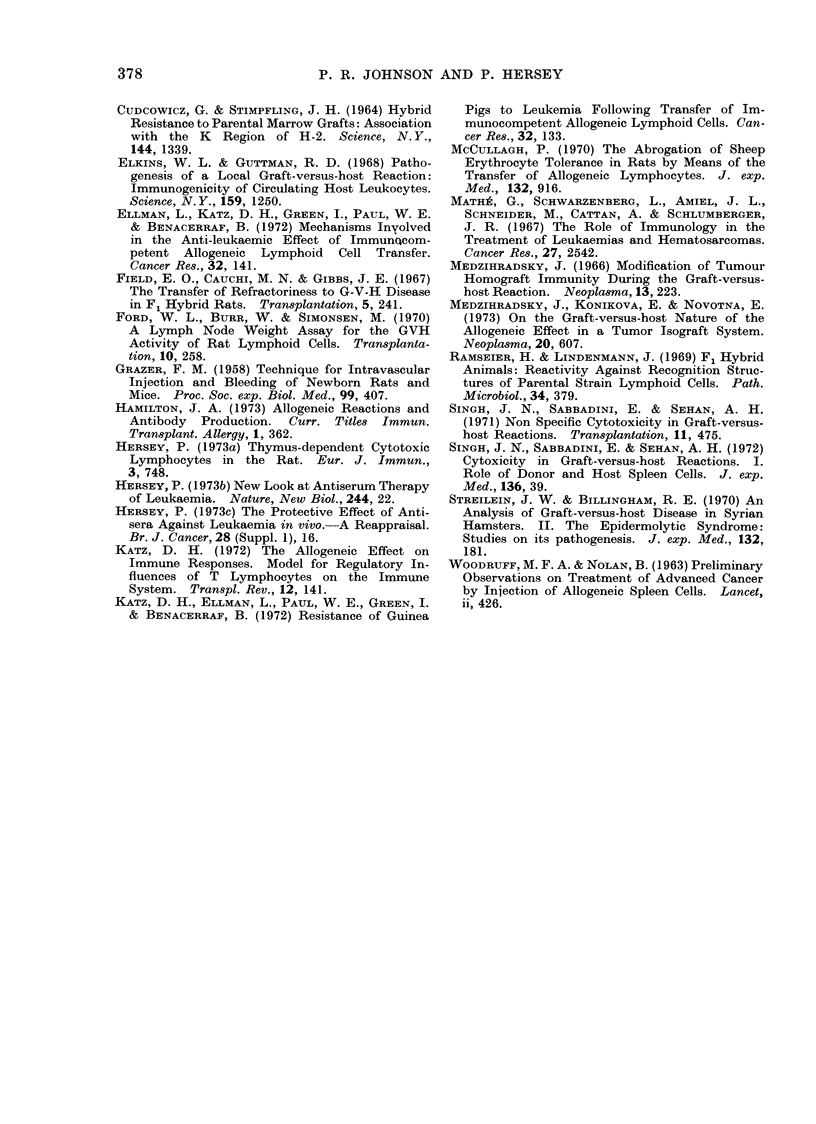


## References

[OCR_00934] Baldwin R. W., Bowen J. G., Price M. R. (1973). Detection of circulating hepatoma D23 antigen and immune complexes in tumour bearer serum.. Br J Cancer.

[OCR_00868] Britton S. (1972). When allogeneic mouse spleen cells are mixed in vitro the T-cells secrete a product which guides the maturation of B-cells.. Scand J Immunol.

[OCR_00885] CUDKOWICZ G., STIMPFLING J. H. (1964). HYBRID RESISTANCE TO PARENTAL MARROW GRAFTS: ASSOCIATION WITH THE K REGION OF H-2.. Science.

[OCR_00891] Elkins W. L., Guttmann R. D. (1968). Pathogenesis of a local graft versus host reaction: immunogenicity of circulating host leukocytes.. Science.

[OCR_00897] Ellman L., Katz D. H., Green I., Paul W. E., Benacerraf B. (1972). Mechanisms involved in the antileukemic effect of immunocompetent allogeneic lymphoid cell transfer.. Cancer Res.

[OCR_00909] Ford W. L., Burr W., Simonsen M. (1970). A lymph node weight assay for the graft-versus-host activity of rat lymphoid cells.. Transplantation.

[OCR_00915] GRAZER F. M. (1958). Technic for intravascular injection and bleeding of newborn rats and mice.. Proc Soc Exp Biol Med.

[OCR_00925] Hersey P. (1973). Thymus-dependent cytotoxic lymphocytes in the rat.. Eur J Immunol.

[OCR_00945] Katz D. H., Ellman L., Paul W. E., Green I., Benacerraf B. (1972). Resistance of guinea pigs to leukemia following transfer of immunocompetent allogeneic lymphoid cells.. Cancer Res.

[OCR_00939] Katz D. H. (1972). The allogeneic effect on immune responses: model for regulatory influences of T lymphocytes on the immune system.. Transplant Rev.

[OCR_00959] Mathé G., Schwarzenberg L., Amiel J. L., Schneider M., Cattan A., Schlumberger J. R. (1967). The role of immunology in the treatment of leukemias and hematosarcomas.. Cancer Res.

[OCR_00953] McCullagh P. J. (1970). The abrogation of sheep erythrocyte tolerance in rats by means of the transfer of allogeneic lymphocytes.. J Exp Med.

[OCR_00971] Medzihradský J., Koníková E., Novotná L. (1973). On the graft-versus-host nature of the allogeneic effect in a tumor isograft system.. Neoplasma.

[OCR_00966] Medzihradský J. (1966). Modification of tumor homograft immunity during the graft-versus-host reaction.. Neoplasma.

[OCR_00977] Ramseier H., Lindenmann J. (1969). F1 hybrid animals: reactivity against recognition structures of parental strain lymphoid cells.. Pathol Microbiol (Basel).

[OCR_00988] Singh J. N., Sabbadini E., Sehon A. H. (1972). Cytotoxicity in graft-versus-host reaction. I. Role of donor and host spleen cells.. J Exp Med.

[OCR_00983] Singh J. N., Sabbadini E., Sehon A. H. (1971). Nonspecific cytotoxicity in graft-versus-host reactions.. Transplantation.

[OCR_00994] Streilein J. W., Billingham R. E. (1970). An analysis of graft-versus-host disease in Syrian hamsters. II. The epidermolytic syndrome: studies on its pathogenesis.. J Exp Med.

[OCR_01001] WOODRUFF M. F., NOLAN B. (1963). PRELIMINARY OBSERVATIONS ON TREATMENT OF ADVANCED CANCER BY INJECTION OF ALLOGENEIC SPLEEN CELLS.. Lancet.

